# Serial Cerebrospinal Fluid Sampling Reveals Trajectories of Potential Synaptic Biomarkers in Early Stages of Alzheimer’s Disease

**DOI:** 10.3233/JAD-240610

**Published:** 2024-08-20

**Authors:** Flora H. Duits, Johanna Nilsson, Henrik Zetterberg, Kaj Blennow, Wiesje M. van der Flier, Charlotte E. Teunissen, Ann Brinkmalm

**Affiliations:** a Alzheimer Center Amsterdam, Neurology, Vrije Universiteit Amsterdam, Amsterdam UMC Location VUmc, Amsterdam, The Netherlands; b Amsterdam Neuroscience, Neurodegeneration, Amsterdam, The Netherlands; c Department of Laboratory Medicine, Neurochemistry Lab, Amsterdam Neuroscience, Amsterdam UMC, Amsterdam, The Netherlands; d Department of Psychiatry and Neurochemistry, Institute of Neuroscience and Physiology, The Sahlgrenska Academy at the University of Gothenburg, Mölndal, Sweden; e Clinical Neurochemistry Laboratory, Sahlgrenska University Hospital, Mölndal, Sweden; f Department of Neurodegenerative Disease and UK Dementia Research Institute, UCL Institute of Neurology, London, UK; g Hong Kong Center for Neurodegenerative Diseases, Hong Kong, China; h Wisconsin Alzheimer’s Disease Research Center, University of Wisconsin School of Medicine and Public Health, University of Wisconsin-Madison, Madison, WI, USA; i Department of Epidemiology and Biostatistics, Amsterdam UMC, Amsterdam, The Netherlands

**Keywords:** Alzheimer’s disease, biomarkers, cognitive decline, mass spectrometry, synapses

## Abstract

**Background:**

Synaptic dysfunction is closely associated with cognitive function in Alzheimer’s disease (AD), and is present already in an early stage of the disease.

**Objective:**

Using serial cerebrospinal fluid (CSF) sampling, we aimed to investigate slopes of CSF synaptic proteins, and their relation with cognition along the AD continuum.

**Methods:**

We included subjects with subjective cognitive decline (SCD) or mild cognitive impairment (MCI) (*n* = 50 amyloid-β+ [*A* +], *n* = 50 A–) and 50 patients with AD dementia from the Amsterdam dementia cohort, with CSF at two time points (median[IQR] 2.1[1.4–2.7] years). We analyzed 17 synaptic proteins and neurofilament light (NfL). Using linear mixed models we assessed trajectories of protein levels, and associations with cognitive decline (repeated Mini-Mental State Examination). We used Cox regression models to assess predictive value of protein levels for progression to AD dementia.

**Results:**

At baseline most proteins showed increased levels in AD dementia compared to the other groups. In contrast NPTX2 levels were lower in AD dementia. Higher baseline levels of SNAP25, β-syn, and 14-3-3 proteins were associated with faster cognitive decline (St.B[SE] –0.27[0.12] to –0.61[0.12]). Longitudinal analyses showed that SYT1 and NPTX levels decreased over time in AD dementia (st.B[SE] –0.10[0.04] to –0.15[0.05]) and SCD/MCI-A+ (St.B[SE] –0.07[0.03] to –0.12[0.03]), but not in SCD/MCI-A- (p_interaction_ < 0.05). Increase over time in NfL levels was associated with faster cognitive decline in AD dementia (St.B[SE] –1.75[0.58]), but not in the other groups (p_interaction_ < 0.05).

**Conclusions:**

CSF synaptic proteins showed different slopes over time, suggesting complex synaptic dynamics. High levels of especially SNAP-25 may have value for prediction of cognitive decline in early AD stages, while increase in NfL over time correlates better with cognitive decline in later stages.

## INTRODUCTION

In the last decade much progress has been made in the search for and validation of diagnostic biomarkers for Alzheimer’s disease (AD).^1^ Biomarker evidence for amyloid-β (Aβ) and tau pathology is now used for a diagnosis of AD, ^2^ and a prerequisite for (inclusion in trials of) disease-modifying treatments.^3 - 5^ However, within individuals with AD there is biological heterogeneity and variability in disease progression, which makes interpreting trial results and prognostication on an individual level difficult.^6,7^ It is still unclear which pathophysiological processes drive this variability. As current disease-modifying therapies do not stop disease progression, the aim is to develop treatment for AD for disease processes other than amyloid pathology. Therefore, understanding these disease processes is of major importance.

Synaptic dysfunction has a central role in AD pathophysiology: it is considered to be closely associated with cognitive function and is present already in an early stage of the disease.^8,9^ The synaptic proteins cerebrospinal fluid (CSF) neurogranin (Ng) and synaptosomal-associated protein 25 (SNAP-25) have been most extensively studied; their concentrations are higher in AD compared with control subjects, and higher levels correlate with faster disease progression.^10 - 17^ Other synaptic proteins are also emerging as potential biomarkers for AD. In previous studies levels of neuronal pentraxins (NPTX) were decreased, while synaptotagmin-1 (SYT1) levels were increased in AD compared to controls.^13,16,18 - 20^ Previously, we found that CSF levels of proteins involved in synapse function were specifically elevated in mild cognitive impairment (MCI)-AD patients, while levels in controls and AD dementia patients were similar to each other.^21^ Neurofilament light (NfL) is a marker for axonal damage, assumed to be downstream of amyloid pathology.^22^ However, it is unclear whether synaptic proteins and NfL have different associations with cognitive decline. Studies with longitudinal CSF data are sparse. Increasing Ng levels in cognitively unimpaired subjects over time, and decreasing levels of SNAP-25, Ng and NPTX2 in AD patients over time have been found.^11,23,24^

In the current study, we aimed to gain a better understanding of synaptic dysfunction in AD by studying a panel of synaptic proteins (including SNAP-25, SYT1, NPTX1 and -2, β- and *γ*-synuclein, 14-3-3 proteins) and NfL, in a longitudinal design, by use of serial CSF sampling in a cohort of memory clinic patients.^25^ We investigated whether changes over time differed between AD stages and whether protein concentrations were associated with cognitive decline.

## MATERIALS AND METHODS

### Study population

We included subjects with subjective cognitive decline (SCD), MCI, and AD dementia (total *n* = 150) from the Amsterdam dementia cohort (ADC),^26^ based on availability of CSF at two time points with an interval of at least one year (median[IQR] 2.1[1.4–2.7] years), and clinical follow-up of at least one year (median[IQR] 3.3[2.1–5.1] years). The ADC is a memory clinic based cohort, where all subjects are included after an extensive baseline visit at the memory clinic. This includes extensive cognitive screening, with physical and neurological examination, EEG, MRI, and laboratory tests including lumbar puncture. Neuropsychological investigation includes at least one test per cognitive domain, as well as Mini-Mental State Examination (MMSE) for global cognition. At standardized annual follow-up consultations, subjects see a clinician and a neuropsychologist, with repeated MMSE and neuropsychological investigation. For this study, subjects were included with available CSF at baseline (*n* = 113 at their first visit, *n* = 37 within 6 months of their first visit), and had a repeated lumbar puncture for research purposes, at one of these consultations (*n* = 117), or at a visit in between (*n* = 33). At each follow-up consultation physical, neurological, and neuropsychological examinations were repeated.

Diagnoses were made by consensus in a multidisciplinary team. AD dementia and MCI were diagnosed according to international criteria in force at the time of inclusion; the criteria of the National Institute of Neurological and Communicative Disorders and Stroke-Alzheimer’s Disease and Related Disorders Association (NINCDS-ADRDA) for AD dementia, the Petersen’s criteria for MCI,^27,28^ or core clinical NIA-AA criteria for AD dementia or MCI.^29,30^ When all investigations were normal (i.e., criteria for MCI or any psychiatric or neurological disorder not fulfilled), patients were labeled as having SCD. All AD dementia patients (*n* = 50) were confirmed with CSF AD biomarkers. The clinical diagnosis of SCD or MCI was determined independent of CSF biomarker results. For this study, SCD and MCI subjects were grouped according to CSF Aβ_42_ abnormality, i.e., SCD/MCI-A- (*n* = 50) and SCD/MCI-A+ (*n* = 50). For biomarker abnormality we used previously published cut-offs.^31,32^ All subjects gave written informed consent for the use of their clinical data and CSF for research purposes. The study was approved by the ethical review board of the Amsterdam University Medical Center, and was in accordance with the Helsinki Declaration of 1975.

### CSF samples and analysis of core AD biomarkers

CSF was obtained by lumbar puncture (LP) using a 25-gauge needle and a syringe and collected in 10 mL polypropylene tubes (Sarstedt, Nümbrecht, Germany). At baseline, part of the CSF was used for routine analysis including leukocyte and erythrocyte count, glucose concentration, and total protein concentration. Within two hours, the remaining CSF was centrifuged at 1800 g for 10 min at 4°C, transferred to new polypropylene tubes and stored either at –20°C until analysis of Aβ_42_, tau and p-tau181, or directly at –80°C in the biobank until further analysis, according to international consensus guidelines.^33,34^ At follow-up, all obtained CSF was centrifuged and directly stored at –80°C in the biobank. Baseline CSF levels of Aβ_42_, total tau and p-tau were measured with commercially available ELISAs (β-amyloid_ (1 -42) _, hTAU-Ag and Phosphotau_ (181P) _; Fujirebio, Ghent, Belgium) on a routine basis as described before.^35^ Measurements took place consecutively within one month of the patient’s baseline visit. Intra-assay coefficients of variation (CV) were (mean±SD) 2.0±0.5% for Aβ_42_, 3.2±1.3% for tau and 2.9±0.8% for p-tau, inter-assay CVs (mean±SD) were 10.9±1.8% for Aβ_42_, 9.9±2.1% for tau and 9.1±1.8% for p-tau. As it is known that Aβ_42_ levels have increased over time as measured with the assay used in our cohort, we used rescaled values of Aβ_42_ as developed previously by our group.^32^ For biomarker abnormality we used previously published cut-offs.^31,32^

### Analysis of synaptic proteins and NfL

Synaptic proteins were analyzed using two different, in-house developed mass spectrometry-based assays described in detail before.^17 - 19^ SYT1 and SNAP-25 were analyzed in one panel, the other proteins were analyzed as a synaptic panel in another (see Supplementary Table 1 for details). Both assays were run on four different plates, with six replicate quality control samples per plate to assess CV. For the proteins for which more than one peptide was analyzed, the peptide with the best analytical performance (lowest CV) was selected for further analyses. Interassay CVs were all below 8%, except for 14-3-3μ (CV of 15%). Intra-assay CVs were all below 15%, only 14-3-3μ had a CV of 24%. NfL concentration was measured on the Single molecule array (Simoa) HD-1 Analyzer (Quanterix, Billerica, MA), using the commercially available NF-Light kit, according to the manufacturer’s instructions. Statistical analysis

For statistical analysis we used SPSS 28 (IBM for Windows) or R (version 4.4.1, ‘Race for your life’). We assessed differences in patient characteristics using chi-squared test, Student’s T-test, or ANOVA when appropriate. For analysis of group differences in baseline levels of synaptic proteins, change in protein levels over time, and association of protein levels with cognitive decline over time we used linear mixed models. This analysis accounts for within-subject correlations over time, is suitable for varying time intervals between assessments, and allows different numbers of assessments per subject. Therefore this method has increased statistical power, as all assessments can be used in the analysis. Each protein was assessed separately. In all models, subject specific random intercepts and random slopes with time were assumed, meaning that the model accounted for individual variation of baseline measure and individual variation of change in outcome measure over time. To obtain standardized beta’s (st.B) of the effects, all proteins were transformed to z-scores, as calculated by (concentration –mean)/SD, based on all measurements (baseline and follow-up). In the first analysis we assessed baseline levels and change in protein levels over time and whether this was dependent on disease stage. We entered protein as dependent variable, and baseline group (SCD/MCI-A–, SCD/MCI-A+ or AD dementia) and time as main independent variables. Interaction group*time was included to determine slopes for each group. Groups were recoded to assess main effects of time per group. Baseline age, sex, and plate of MS analysis were entered as covariates. In the second analysis we assessed associations of baseline protein levels with cognitive decline (measured by repeated MMSE). We entered MMSE as dependent variable, and protein and time as independent variables. The interaction protein*time was included to determine the association of protein level with rate of cognitive decline, and protein*group*time to assess differences in this slope between the groups. For significant third-degree interactions we calculated betas per group. Baseline age, sex, level of education (Verhage scores^36^) and age*time were entered as covariates. We repeated this analysis with annual change in protein levels (in z-scores) instead of baseline levels, to assess association of change in protein levels with cognitive decline. Lastly, we assessed predictive value of the proteins for progression to AD dementia from the stage of SCD or MCI, using Cox proportional hazards models. First, each protein was assessed in a univariate analysis (dichotomized at the median value of the total cohort). We used three different models. In the first model only the protein was entered, in the second model we adjusted for baseline age and sex, and in the third model we added Aβ-positivity (dichotomized) as additional covariate. As sensitivity analysis, we repeated the analyses after excluding Aβ-negative subjects. Proteins with significant effects in the univariate analysis were included in a multivariate analysis using backward stepwise selection to assess combined predictive value, first with only these proteins, baseline age and sex. As a second step we included ATN classification (based on in-house CSF biomarker cut-offs for Aβ_42_ [A], p-tau181 [T], and total tau [N]), to assess added value of the synaptic proteins if combined with the core AD biomarkers. Predictive values of the different proteins are presented as hazard ratios (HRs) with 95% CIs. Statistical significance was set at *p* < 0.05 for main effects and at *p* < 0.10 for interaction terms.

## RESULTS

### Baseline characteristics

Baseline characteristics are shown in Table 1. SCD/MCI-A– subjects were slightly younger and less often female compared to the other two groups. The interval between baseline and follow-up LP was longer in SCD/MCI-A– subjects compared to AD dementia patients, as was the total clinical follow-up time. Baseline MMSE values were lower in SCD/MCI-A+ compared to the A– subjects, and as expected lowest in AD dementia patients. Annual change in MMSE was negligible in A– subjects and highest in AD dementia patients. By definition, levels of CSF Aβ_42_, tau and p-tau differed between groups.

**Table 1:** Patient characteristics

	SCD/MCI-A–	SCD/MCI-A+	AD dementia
	(*n* = 50)	(*n* = 50)	(*n* = 50)
Age	61 (7)	68 (7)^***^	64 (7)^$^
Sex (F, %)^a^	19 (32%)	19 (48%)	24 (48%)
LP interval	3.2 (2.2)	2.7 (1.8)	1.9 (0.9)^***^
Years of follow-up	5.1 (3.3)	4.1 (2.2)	2.9 (1.5)^***^
MMSE at baseline	28 (2)	27 (2)	23 (4)^$$$***^
Annual MMSE change	0 (0.7)	–1.0 (1.4)^**^	–2.0 (2.2)^$$***^
Aβ_42_ (A)	1134 (196)	643 (104)^***^	613 (104)^***^
pTau-181 (T)	46 (15)	82 (35)^***^	99 (38)^$***^
T-tau (N)	280 (122)	585 (313)^***^	806 (427)^$$***^

Mean±SD unless otherwise stated. aShown are n (proportion of total within diagnostic group). ^*^p < 0.05 versus SCD/MCI-A– subjects. ^**^p < 0.01 versus SCD/MCI-A– subjects. ^***^p < 0.001 versus SCD/MCI-A– subjects. ^$^*p* < 0.05 versus SCD/MCI-A+ subjects. ^$$^*p* < 0.01 versus SCD/MCI-A+ subjects. ^$$$^*p* < 0.001 versus SCD/MCI-A+ subjects.

### Differences between groups in baseline CSF protein levels and slopes over time

**Fig. 1:**
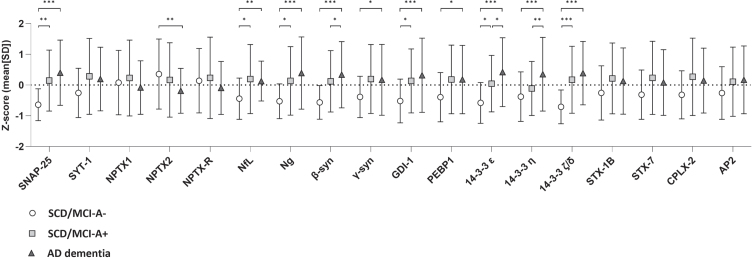
Baseline levels of synaptic proteins per group. Shown are mean Z-scores±SD, *p*-values were calculated with the linear mixed model, adjusted for age, sex and MS plate (as experiments were performed on four different plates). All biomarkers were included as dependent variable in separate models, with group, time and interaction time*group as independent variables. The main effect of group represents differences in baseline levels between groups. ^*^p < 0.05; ^**^p < 0.01; ^***^p < 0.001.

**Fig. 2:**
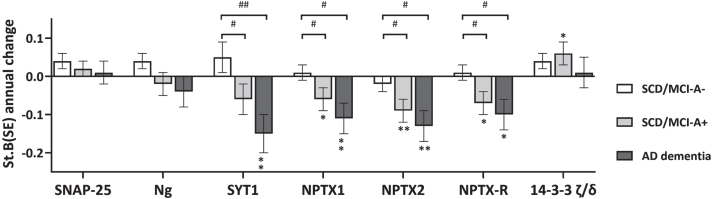
Change in levels of synaptic proteins over time. Shown are standardized betas (SE) of annual change in protein levels for a selection of proteins, as calculated with the linear mixed model, adjusted for age, sex and MS plate (as experiments were performed on four different plates). All biomarkers were included as dependent variable in separate models, with group, time and interaction time*group as independent variables. The main effect of time represents annual change in biomarker levels, the interaction term represents differences between groups in change over time. Groups were recoded to assess main effects of time per group. ^*^p < 0.05 versus slope zero; ^**^p < 0.01 versus slope zero. ^#^*p* < 0.05 versus SCD/MCI-A-; ^##^*p* < 0.01 versus SCD/MCI-A-.

Results of baseline differences (i.e. main effect of group) are shown in Fig. 1 and Supplementary Table 2. All 14-3-3 proteins, SNAP-25, Ng, β-syn, and GDI- showed lowest levels in SCD/MCI-A– subjects and highest levels in AD dementia, with levels of SCD/MCI-A+ subjects in between. NfL, *γ*-syn, and PEBP-1 showed equally increased levels in SCD/MCI-A+ subjects and AD dementia patients compared to SCD/MCI-A– subjects. In contrast, levels of all three NPTX proteins at baseline were lower in AD dementia patients compared to both groups of SCD/MCI subjects, although only in NPTX2 this was significant (main effect of group *p* < 0.05). Longitudinal changes (main effect of time) are shown in Fig. 2 and Supplementary Table 2. Levels of SYT1 and all NPTX proteins decreased, while 14-3-3 *ζ*/*δ* increased over time (*p* < 0.05 for main effect of time). There was a significant interaction with group for SYT1, NPTX1, NPTX2, and NPTX-R; slopes of AD dementia patients (st.B[SE] –0.15[0.05], –0.11[0.04], –0.13 [0.04], and –0.10[0.04] respectively) differed from those of SCD/MCI-A– subjects. Slopes of SCD/MCI-A+ subjects (st.B[SE] – 0.06[0.04], – 0.06[0.03], – 0.09[0.03], and –0.07[0.03] respectively), were in between those of A– subjects and AD dementia patients. 14-3-3 *ζ*/*δ* increased most in SCD/MCI-A+ subjects (st.B[SE] 0.06[0.03], *p* < 0.05), but the interaction time*group was not significant. The other proteins showed no change over time nor difference in slopes between the groups. See Supplementary Figure 1 for slopes over time of all protein levels.

### Association with cognitive decline

**Fig. 3:**
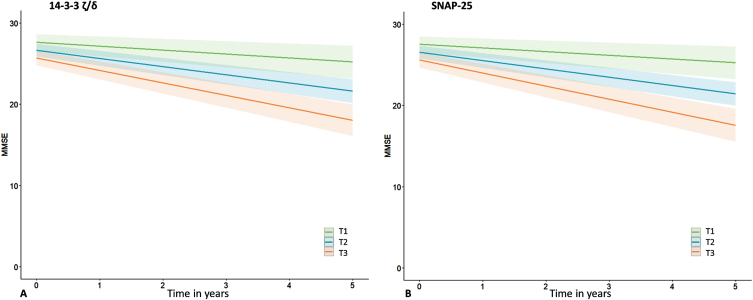
Associations with cognitive decline. Shown are associations between synaptic proteins (14-3-3 *ζ*/*δ* and SNAP-25) and cognitive decline (slopes in MMSE over time), estimated with linear mixed models. Subject specific random intercepts and random slopes with time were assumed. Age, sex, and level of education were entered as covariates. Analyses were performed with continuous values for the synaptic proteins, tertiles were created for visualization purposes only.

**Fig. 4:**
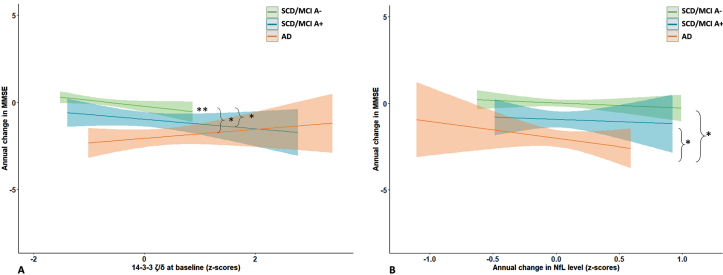
Associations with cognitive decline: differences between groups. Shown are differences between baseline groups in association of baseline 14-3-3 *ζ*/*δ* (A) and change in NfL (B) with cognitive decline, estimated with linear mixed models. Subject specific random intercepts and random slopes with time were assumed. Age, sex, and level of education were entered as covariates. Differences between groups were estimated using the interaction group*protein*time.

Next, we assessed whether baseline and changes in protein levels were associated with cognitive decline as measured by repeated MMSE, and whether this association was disease stage dependent. Detailed results are shown in Supplementary Table 3. In the total cohort MMSE declined with – 0.9(0.1) points per year (B[SE]), ranging from 0.0(0.1) in the SCD/MCI-A– subjects to –2.0(0.2) in the AD dementia patients. Without adjustment for group (i.e., in the total cohort), there were main effects of SNAP-25, Ng, β-syn, NPTX2, GDI-1, and all three 14-3-3 proteins. Most betas were negative, indicating higher baseline levels were associated with lower baseline MMSE. The associations were strongest for SNAP-25 (St.B[SE] – 1.00[0.28], *p* < 0.001), and 14-3-3 *ζ*/*δ* (–0.99[0.28], *p* < 0.001). In contrast, for NPTX2 the association was reversed: higher levels were associated with higher baseline MMSE (St.B[SE] 0.68[0.26], *p* < 0.05). Higher baseline levels of most proteins (SNAP-25, NfL, Ng, β-syn, *γ*-syn, AP2-complex subunit β [AP2], GDI-1, PEBP-1, all 14-3-3 proteins and CPLX2) were also associated with faster cognitive decline (interaction protein*time *p* < 0.05). SNAP-25 and 14-3-3 *ζ*/*δ* showed largest effects (St.B[SE] – 0.61[0.12] and –0.60[0.12] respectively, both *p* < 0.001; Fig. 3A, B). In line with the association at baseline, higher level of NPTX2 was associated with a slower rate of cognitive decline (St.B[SE] 0.22[0.11], *p* < 0.05). For interaction effects (protein*time) of all proteins see Supplementary Figure 2. Next, we included group as main effects and the interactions with protein and time in the model. For NfL and 14-3-3 *ζ*/*δ* the association with cognitive decline differed between SCD/MCI A– and AD dementia (interaction group*protein*time *p* < 0.10). Stratified analysis showed for both proteins that higher levels were associated with faster cognitive decline in the SCD/MCI A– group (St.B[SE] – 0.26[0.08], *p* < 0.01 for NfL, –0.38[0.11], *p* < 0.01 for 14-3-3 *ζ*/*δ*), but not in the other two groups. Figure 4A shows visualization of the slopes for 14-3-3 *ζ*/*δ*. For the other proteins there were no third-degree interactions, indicating that associations between baseline protein levels and cognitive decline were not different between the groups.

We repeated the analysis with annual change in protein levels in the model instead of baseline levels, detailed results are shown in Supplementary Table 4. NfL showed an association specifically in the AD dementia group: increase in NfL levels over time was associated with lower baseline MMSE (B[SE] – 3.44[1.61]) and faster decline in MMSE over time (–1.75[0.58], *p* < 0.01; *p* = 0.06 for interaction protein*group*time; Fig. 4B). For SNAP-25 there was a trend for an association between annual increase in protein level and cognitive decline (B[SE] – 0.91[0.47], *p* = 0.06), but the interaction protein*group*time was not significant. Decrease in levels of NPTX proteins or SYT1 was not associated with cognitive decline, although for NPTX2 there was a trend for an association in the total cohort (B[SE] 0.67[0.40], *p* < 0.10).

### Prediction of progression to AD dementia

For subjects without dementia at baseline (*n* = 100) we assessed whether levels of synaptic proteins and NfL, dichotomized at the median value of all baseline measurements, were associated with disease progression. In the unadjusted model nine proteins were associatied with disease progression (Table 2), and SNAP-25 showed the largest effect (HR[95% CI] 4.9[2.4–9.7]). After correction for age and sex (Model 2) six proteins remained significant predictors, with SNAP-25 showing the largest HR (4.4 [2.1–9.1]). After we added Aβ-positivity (dichotomous) as covariate to the model (Model 3), HRs were somewhat attenuated. When we included only *A* + subjects in the analysis, effects were roughly similar compared to Model 3. In the multivariate analysis using backward stepwise selection, the final model included only age (HR[95% CI] 1.09[1.04–1.14], *p* < 0.001) and SNAP-25 (HR[95% CI] 3.6[1.8–7.3], *p* < 0.001; Table 3). When ATN classification was added to the model, this had the largest effect (*p* < 0.01), with the largest HR for the group with all three markers abnormal (7.2 [1.6–32.5]). However, SNAP-25 was still a significant predictor in this model (HR[95% CI] 2.6[2.0–6.9], *p* < 0.05; Table 3).

**Table 2:** Univariate cox proportional hazards models

Variable ^a^	Model 1	Model 2	Model 3	Model 3
				Only *A*+
SNAP-25	4.9 (2.4–9.7)	4.4 (2.1–9.1)	3.8 (1.8–8.2)	3.1 (1.3–7.2)
NfL	4.5 (2.2–9.3)	2.7 (1.1–6.5)	2.1 (0.9–5.0)	2.0 (0.8–4.9)
14-3-3 *ζ*/*δ*	4.1 (2.1–8.2)	2.9 (1.4–5.9)	3.1 (1.5–6.3)	3.1 (1.4–6.9)
β-synuclein	3.6 (1.8–7.1)	2.5 (1.2–5.2)	3.0 (1.4–6.1)	2.8 (1.2–6.2)
Ng	3.2 (1.6–6.1)	2.5 (1.2–5.0)	3.0 (1.4–6.3)	3.1 (1.3–7.2)
14-3-3 *ɛ*	2.8 (1.5–5.4)	2.4 (1.2–4.9)	3.0 (1.4–6.4)	2.5 (1.1–5.7)
GDI-1	2.6 (1.3–5.0)	1.8 (0.9–3.5)	2.1 (1.0–4.2)	1.9 (0.9–4.1)
PEBP1	2.4 (1.2–4.6)	1.7 (0.8–3.3)	2.2 (1.1–4.4)	2.1 (1.0–4.7)
*γ*-synuclein	2.0 (1.0–3.8)	1.3 (0.7–2.7)	1.6 (0.8–3.4)	1.5 (0.7–3.3)
14-3-3 *η*	1.8 (0.9–3.4)	1.8 (0.9–3.4)	2.2 (1.1–4.4)	2.3 (1.0–5.0)
STX1B	1.7 (0.9–3.2)	1.2 (0.6–2.4)	1.9 (0.9–3.8)	1.9 (0.9–4.1)
STX7	1.8 (0.9–3.5)	1.3 (0.7–2.5)	1.8 (0.9–3.7)	1.9 (0.9–4.0)
CPLX2	1.8 (0.9–3.4)	1.4 (0.7–2.7)	2.2 (1.1–4.5)	2.3 (1.1–5.0)
NPTX1	1.2 (0.6–2.3)	1.1 (0.6–2.2)	1.8 (0.9–3.5)	1.7 (0.8–3.6)
NPTX2	1.2 (0.6–2.2)	1.0 (0.5–2.0)	0.6 (0.3–1.2)	0.6 (0.3–1.3)
NPTX-R	1.1 (0.6–2.2)	1.1 (0.6–2.2)	1.8 (0.9–3.5)	1.7 (0.8–3.6)

Shown are HR (95% CI) for disease progression (MCI-AD in SCD subjects or AD dementia in MCI subjects). ^a^all proteins dichotomized at median value of all baseline values. Model 1: without correction. Model 2: with correction for age and sex. Model 3: with addition of Aβ positivity (dichotomized).

**Table 3:** Multivariate cox proportional hazards models

Variable	Model 1	Model 2
	Only synaptic	With ATN
	proteins	classification
Age ^a^	1.09 (1.04–1.14)^***^	1.07 (1.01–1.13)^*^
A–T–N–^b^	n/a	REF
A–T+/N+ ^b^	n/a	0.7 (0.1–4.0)
A+T–N–^b^	n/a	4.4 (1.0–19.0)^*^
A+T+/N+ ^b^	n/a	3.2 (0.5–19.5)
A+T+N+ ^b^	n/a	7.2 (1.6–32.5)^**^
SNAP– 25 ^c^	3.6 (1.8–7.3)^***^	2.6 (1.0–6.9)^*^

Shown are HR (95% CI) for disease progression (MCI-AD in SCD subjects or AD dementia in MCI subjects). In the multivariate model backwards stepwise selection was used, only significant predictors are shown. ^a^Age entered as continuous variable. ^b^ATN classification stratified according to in-house cut-offs of CSF biomarkers. ^c^Dichotomized at median value of all baseline values. Model 1: all proteins which showed significant HRs in the univariate models after correction for age and sex were added in this model, in addition to age (in years) and sex (F/M). Model 2: all factors of Model 1 were added and in addition ATN classification (with A-T-N- as reference group). ^*^= *p* < 0.05; ^**^= *p* < 0.01; ^***^= *p* < 0.001.

## DISCUSSION

In this study we investigated longitudinal slopes and prognostic value of a panel of synaptic biomarkers in CSF, with the aim to gain a better understanding of synaptic dysfunction in AD and relation with cognitive decline over time. We found that baseline levels of several synaptic proteins and NfL were higher in MCI subjects with AD pathology and AD dementia patients compared to non-demented subjects with normal Aβ levels. Most of the proteins showing baseline differences between the groups remained stable over time. In contrast, levels of SYT1, NPTX1, and NPTXR were similar between groups at baseline but decreased over time in subjects with AD pathology. Only 14-3-3 *ζ*/*δ* showed higher levels at baseline and increased further, specifically in Aβ-positive non-demented subjects, while NPTX2 showed lower levels at baseline in AD dementia patients and decreased further over time. Second, we showed that especially higher baseline levels of SNAP-25, and in lesser extent of NfL, Ng, β-syn, proteins of the 14-3-3 family, GDI-1 and PEBP-1 were associated with faster cognitive decline and disease progression along the AD continuum. Third, increase over time in levels of SNAP-25 and NfL were associated with faster cognitive decline in AD dementia patients. As the interval between lumbar punctures was shorter than the total clinical follow-up, this implies that increase in these protein levels was associated with future cognitive decline.

Several of our findings at baseline are in line with previous research. Higher levels of SNAP-25 and Ng in patients with prodromal AD and AD dementia, and lower CSF levels of NPTX2 in AD patients compared to controls have been described before.^11 - 17,37,38^ In addition, we replicated previous findings by showing higher levels of synucleins and 14-3-3 proteins in AD compared to cognitively unimpaired subjects.^19,39^ For synaptic biomarkers to be used in trial settings or clinical practice it is of great importance to know if and how levels change over time in the natural disease course, but longitudinal studies are sparse. Decreasing NPTX2, Ng and SNAP-25 levels over time have been described in MCI and AD dementia subjects in the ADNI cohort.^23,24,40^ We previously showed increasing Ng levels in cognitively unimpaired subjects.^11^ For the other synaptic proteins we are not aware of studies with longitudinal designs to assess intra-individual slopes. With our longitudinal design we hence extend these earlier studies, and longitudinal changes we found were in line with previous results of cross-sectional studies. In the current study we found that levels of 14-3-3 *ζ*/*δ* increased over time, and levels of SYT1 and the NPTX proteins decreased over time, while other proteins remained stable. As all of these proteins are expressed within the synapse, this is a remarkable finding; one may assume that their CSF levels would change similarly with accumulating synaptic damage during disease progression. An explanation for these diverging trajectories may lie either in different functions or different location of the proteins in the neuron. A decrease in NPTX protein levels may reflect degeneration of GABA-ergic parvalbumin interneurons, which highly express proteins of the NPTX family and are known to be affected relatively early in AD pathology.^37,41^ Compared to other synaptic proteins therefore, NPTX levels may start to decline earlier during the course of AD. SYT1 is mainly located in synaptic vesicles and is involved in neurotransmitter release at the synapse, while the other proteins are located either in the presynaptic or postsynaptic terminal.^42,43^ A decrease in SYT1 may reflect synaptic dysfunction before major synaptic or neuronal loss. In addition, the interval between lumbar punctures in our study may have been too short to detect small changes over time for the other proteins. Future studies with a higher number of repeated lumbar punctures over a longer time interval along the AD continuum may reveal trajectories of other synaptic proteins in more detail.

We found associations of baseline levels of several proteins with cognitive decline in the total cohort. Specifically in non-demented subjects with normal Aβ levels, higher levels of NfL and 14-3-3 *ζ*/*δ* were associated to faster cognitive decline. In addition, high SNAP-25 levels predicted conversion from MCI to AD dementia, independent of Aβ_42_ and tau status. These results are in line with results of a recent study, where additionally ratios of SNAP-25 and 14-3-3 *ζ*/*δ* with NPTX2 increased predictive value.^39^ As our cohort was relatively small, future studies with a larger sample size are warranted to confidently validate these findings. Furthermore, increase in NfL and SNAP-25 levels over time was associated with faster cognitive decline specifically in AD dementia patients, while on average in the total cohort these proteins did not increase over time. A sharper increase of NfL levels around conversion to dementia has been found in genetic forms of FTD as well.^44^ Previously, decline in NPTX2 levels has been found to be associated with cognitive decline,^24^ but we could not convincingly replicate these results. All combined, our findings suggest that high levels of NfL and synaptic proteins are predictive of cognitive decline in early AD stages, with SNAP-25 as most promising biomarker, and further increase of especially NfL and SNAP-25 levels is associated with faster cognitive decline in the dementia stage of AD. Hence, NfL and SNAP-25 may have added value for individualized prognosis, trial selection and treatment in AD.

A major strength of this study is the availability of a large sample with repeated CSF. As this is difficult to obtain and is known to be a hurdle for patients included in trials,^45^ blood-based biomarkers for synaptic damage could be a solution as they are more easily obtained and repeated. Previously, assessment of Ng in blood has shown no association with levels in CSF, probably because of peripheral synthesis.^46^ However, as β-synuclein and SNAP-25 are highly expressed in brain tissue,^47^ they are interesting targets for the development of a blood-based synaptic biomarker. For β-synuclein a serum assay has been developed recently.^48^ In the current study however, we found highest effect sizes for SNAP-25. Future research is needed to investigate the possibilities for developing a blood-based assay for SNAP-25as well.

Other strengths of this study are the detailed phenotyped patients with consensus diagnosis after each visit to our clinic, and use of sensitive and specific state-of-the-art techniques enabling us to quantify low-abundant proteins. A possible limitation was that we merged the SCD and MCI subjects into combined groups with versus without abnormal Aßlevels. However, separating these groups would have negatively impacted the power, while in exploratory analyses SCD and MCI subjects within each Aßgroup showed mostly similar protein levels. Another limitation may be that clinical follow-up was relatively short for some individuals. Although we have not included subjects who received a diagnosis of another dementia during follow-up in this study, some of the non-AD subjects may have been in a prodromal stage of a non-AD dementia.

In conclusion, we showed diverging longitudinal trajectories of synaptic protein levels over time, and disease-stage dependent associations with cognitive decline. Especially SNAP-25 may have value as either prognostic biomarker in predementia stages of AD, or predictive biomarker in trials, while others such as NPTX-2 or 14-3-3 proteins may serve as surrogate outcome measures in trials, ideally as blood-based biomarkers.^49^

## AUTHOR CONTRIBUTIONS

Flora Duits (Conceptualization; Data curation; Formal analysis; Funding acquisition; Investigation; Methodology; Validation; Visualization; Writing – original draft; Writing – review & editing); Johanna Nilsson (Data curation; Formal analysis; Methodology; Validation; Writing – review & editing); Henrik Zetterberg (Supervision; Writing – review & editing); Kaj Blennow (Conceptualization; Supervision; Writing – review & editing); Wiesje M. van der Flier (Supervision; Writing – review & editing); Charlotte E. Teunissen (Conceptualization; Supervision; Writing – review & editing); Ann Brinkmalm (Data curation; Formal analysis; Methodology; Supervision; Validation; Writing – review & editing).

## Supplementary Material

Supplementary Material

## Data Availability

The data supporting the findings of this study are available on request from the corresponding author. The data are not publicly available due to privacy or ethical restrictions.
