# Supplementary material for: Serial Cerebrospinal Fluid Sampling Reveals Trajectories of Potential Synaptic Biomarkers in Early Stages of Alzheimer’s Disease
Source: J Alzheimers Dis. 2024 Aug 20;100(Suppl 1):S103–14. doi: 10.3233/JAD-240610 (PMC11492063; doi:10.3233/JAD-240610)
Supplement: Supplementary Material [file jad-100-jad240610-s001.pdf]

Supplementary Material

Serial Cerebrospinal Fluid Sampling Reveals Trajectories of Potential Synaptic Biomarkers in Early Stages of Alzheimer's Disease

Supplementary Table 1A. LC-MS/MS settings for the analysis of the synaptic protein panel.

	**Parameter**	**Setting**
**LC**	Sample injection volume	40 µL
	Flow-rate	0.3 mL/min
	Gradient	Broken; 5–20%B (20 min), 20–35%B (7 min)
	Total cycle time	30 min
	Mobile phase A	0.1% formic acid in water (v/v)
	Mobile phase B	0.1% formic acid/84% acetonitrile in water (v/v)
**Electrospray**	Mode	Positive
	Gas temperature	220 °C
	Gas flow	15 L/min
	Nebulizer pressure	40 psi
	Sheath gas temperature	200 °C
	Sheath gas flow	11 L/min
	Capillary voltage	3500 V
	Nozzle voltage	500 V
**iFunnel**	Mode	Positive
	High-pressure radio frequency	200 V
	Low-pressure radio frequency	160 V
**MRM method**	Retention time window	0.8 min
	Collision energies	Individually optimized per transition
	Cell accelerator voltage	Individually optimized per transition

Supplementary Table 1B. LC-MS/MS settings for the analysis of SNAP-25 and Synapotagmin-1.

	Parameter	Setting
**LC**	Sample injection volume	40 µL
	Flow-rate	0.3 mL/min
	Gradient	Broken; 0–16%B (0.1 min), 16–19%B (11 min), 19–100%B (0.4 min)
	Total cycle time	12.5 min
	Mobile phase A	0.1% formic acid in water (v/v)
	Mobile phase B	0.1% formic acid/84% acetonitrile in water (v/v)
**Electrospray**	Mode	Positive
	Gas temperature	220 °C
	Gas flow	15 L/min
	Nebulizer pressure	30 psi
	Sheath gas temperature	200 °C
	Sheath gas flow	11 L/min
	Capillary voltage	4000 V
	Nozzle voltage	300 V
**iFunnel**	Mode	Positive
	High-pressure radio frequency	120 V
	Low-pressure radio frequency	100 V
**MRM method**	Retention time window	1 min
	Collision energies	Individually optimized per transition
	Cell accelerator voltage	2 V

Supplementary Table 2. Baseline levels and change over time per group

	Total cohort	SCD/MCI- (n=50)	SCD/MCI+ (n=50)	AD dementia (n=50)	p main effect	$p_{interaction}$ group*time
Baseline SNAP-25		-0.64 (0.52)	0.15 (0.99)$^{\#\#}$	0.40 (1.06)$^{\#\#\#}$	<0.001	
Annual change	0.03 (0.01)	0.04 (0.02)	0.02(0.02)	0.01 (0.03)	0.13	0.82
Baseline SYT1		-0.26 (0.80)	0.28 (1.23)^{1}	0.20 (1.04)^{1}	0.09	
Annual change	-0.04 (0.02)*	0.05 (0.04)	-0.06 (0.04)$^{\#}$	-0.15 (0.05)**$^{\#\#}$	<0.05	<0.01
Baseline NfL		-0.44 (0.67)	0.19 (1.12)$^{\#}$	0.13 (0.65)$^{\#\#}$	<0.001	
Annual change	0.05 (0.03)	0.06 (0.05)	0.04 (0.06)	0.02 (0.07)	0.24	0.88
Baseline Ng		-0.53 (0.56)	0.13 (1.12)$^{\#}$	0.39 (1.17)$^{\#\#\#}$	<0.001	
Annual change	0.00 (0.02)	0.04 (0.02)	-0.02 (0.03)	-0.04 (0.05)	0.93	0.26
Baseline β-syn		-0.57 (0.55)	0.12 (1.00)	0.34 (1.08)$^{\#\#\#\$}$	<0.001	
Annual change	0.03 (0.02)	0.04 (0.02)*	-0.01 (0.03)	0.06 (0.04)	0.08	0.26
Baseline γ-syn		-0.39 (0.67)	0.20 (1.12)	0.17 (1.15)$^{\#}$	<0.05	
Annual change	0.02 (0.02)	0.05 (0.03)	0.00 (0.03)	0.01 (0.05)	0.37	0.47
Baseline AP2		-0.26 (0.86)	0.11 (1.13)	0.17 (1.10)	0.22	
Annual change	0.00 (0.02)	0.03 (0.03)	-0.03 (0.03)	-0.04 (0.05)	0.57	0.25
Baseline NPTX1		0.08 (1.05)	0.23 (1.23)	-0.08 (0.87)	0.50	
Annual change	-0.04 (0.02)*	0.01 (0.02)	-0.06 (0.03)*$^{\#}$	-0.11 (0.04)**$^{\#}$	<0.01	<0.05
Baseline NPTX2		0.36 (1.14)	0.17 (1.21)	-0.19 (0.73)$^{\#\#}$	<0.05	
Annual change	-0.06 (0.02)**	-0.02 (0.02)	-0.09 (0.03)**$^{\#}$	-0.13 (0.04)**$^{\#}$	<0.001	<0.05
Baseline NPTX-R		0.14 (1.05)	0.23 (1.32)	-0.09 (0.86)	0.34	
Annual change	-0.04 (0.02)*	0.01 (0.02)	-0.07 (0.03)*$^{\#}$	-0.10 (0.04)*$^{\#}$	<0.01	<0.05
Baseline GDI-1		-0.52 (0.71)	0.13 (1.04)$^{\#}$	0.32 (1.21)$^{\#\#\#}$	<0.001	
Annual change	0.02 (0.02)	0.05 (0.03)	0.03 (0.03)	-0.03 (0.05)	0.47	0.43
Baseline PEBP1		-0.39 (0.80)	0.18 (1.12)	0.18 (1.11)$^{\#}$	<0.05	
Annual change	0.02 (0.02)	0.05 (0.03)	0.02 (0.03)	-0.03 (0.05)	0.72	0.27
Baseline 14-3-3 ε		-0.58 (0.66)	0.04 (0.92)$^{\#}$	0.42 (1.12)$^{\#\#\#\$}$	<0.001	
Annual change	0.03 (0.02)	0.05 (0.03)	0.03 (0.03)	0.02 (0.05)	0.15	0.88
Baseline 14-3-3 η		-0.38 (0.81)	-0.11 (0.88)	0.35 (1.20)$^{\#\#\#\$\$}$	<0.001	
Annual change	0.03 (0.02)	0.03 (0.04)	0.05 (0.04)	0.03 (0.06)	0.21	0.90
Baseline 14-3-3 ζ/δ		-0.71 (0.55)	0.17 (1.09)$^{\#\#\#}$	0.39 (1.03)$^{\#\#\#}$	<0.001	
Annual change	0.04 (0.02)*	0.04 (0.02)	0.06 (0.03)*	0.01 (0.04)	<0.05	0.65
Baseline STX-1B		-0.26 (0.88)	0.21 (1.15)	0.13 (1.08)	0.28	
Annual change	0.00 (0.02)	0.04 (0.03)	-0.03 (0.03)	-0.07 (0.05)^{1}	0.32	0.09
Baseline STX-7		-0.31 (0.80)	0.23 (1.19)	0.08 (1.07)	0.20	
Annual change	0.02 (0.02)	0.05 (0.03)	0.00 (0.03)	-0.04 (0.05)	0.91	0.19
Baseline CPLX-2		-0.32 (0.78)	0.27 (1.26)	0.15 (1.06)	0.14	
Annual change	-0.01 (0.02)	0.01 (0.02)	-0.03 (0.03)	-0.04 (0.04)	0.35	0.38

In the table baseline values are crude mean Z-scores ± standard deviations, p-values were calculated with the linear mixed model, adjusted for age, sex, and MS plate. Annual change is shown as St.B (SE), as derived from the linear mixed model. All biomarkers were included as dependent variable in separate models, with group, time and interaction time*group as independent variables. The main effect of group represents baseline protein level per group, differences between groups were assessed with post-hoc tests and Bonferroni corrections. The main effect of time represents annual change in protein levels per group, the interaction term represents differences between groups in change over time. Groups were recoded to assess main effects of time per group.

* p<0.05; ** p<0.01; *** p<0.001 (slope different from zero)

$^{\$}$ p<0.05 versus SCD/MCI+; $^{\$\$}$ p<0.01 versus SCD/MCI+; $^{\$\$\$}$ p<0.001 versus SCD/MCI+

$^{\#}$ p<0.05 versus SCD/MCI-; $^{\#\#}$ p<0.01 versus SCD/MCI-; $^{\#\#\#}$ p<0.001 versus SCD/MCI-

^{1} p<0.10 versus SCD/MCI-

Supplementary Table 3. Association of baseline protein levels with baseline MMSE and cognitive decline

	Total cohort	SCD/MCI- (n=50)	SCD/MCI+ (n=50)	AD dementia (n=50)	$P_{interaction}$ prot*time*group
Main effect SNAP-25	-1.00 (0.28)***				
SNAP-25*time	-0.61 (0.12)***				0.47
Main effect SYT1	-0.02 (0.27)				
SYT1*time	-0.21 (0.12)				0.54
Main effect NfL	-0.60 (0.33)				
NfL*time	-0.37 (0.15)*	-0.44 (0.22)*	-0.04 (0.15)	0.14 (0.24)[#]	0.08
Main effect Ng	-0.63 (0.27)*				
Ng*time	-0.43 (0.12)***				0.44
Main effect β-syn	-0.87 (0.28)**				
β-syn*time	-0.53 (0.12)***				0.57
Main effect γ-syn	-0.31 (0.28)				
γ-syn*time	-0.27 (0.12)*				0.27
Main effect AP2	-0.27 (0.27)				
AP2*time	-0.27 (0.12)*				0.55
Main effect NPTX1	0.42 (0.27)				
NPTX1*time	0.09 (0.11)				0.29
Main effect NPTX2	0.68 (0.26)**				
NPTX2*time	0.22 (0.11)*				0.35
Main effect NPTX-R	0.48 (0.27)				
NPTX-R*time	0.10 (0.12)				0.48
Main effect GDI-1	-0.60 (0.27)*				
GDI-1*time	-0.46 (0.12)***				0.31
Main effect PEBP1	-0.28 (0.28)				
PEBP1*time	-0.33 (0.12)**				0.36
Main effect 14-3-3 ε	-0.88 (0.28)***				
14-3-3 ε*time	-0.53 (0.12)***				0.13
Main effect 14-3-3 η	-0.71 (0.28)**				
14-3-3 η*time	-0.42 (0.12)***				0.51
Main effect 14-3-3 ζ/δ	-0.99 (0.28)***				
14-3-3 ζ/δ*time	-0.60 (0.12)***	-0.70 (0.26)**	-0.17 (0.14)[#]	-0.16 (0.17)[#]	0.08
Main effect STX1-B	-0.14 (0.28)				
STX-1B*time	-0.23 (0.12)				0.41
Main effect STX-7	-0.05 (0.28)				
STX-7*time	-0.16 (0.12)				0.34
Main effect CPLX-2	-0.12 (0.27)				
CPLX-2*time	-0.25 (0.12)*				0.39

Proteins were all transformed to Z-scores and evaluated in separate models. Estimates for the total cohort were calculated without a term for group. The main effect of each protein represents the association between baseline protein level and baseline MMSE, the interaction protein*group was entered to assess whether main effects of proteins differed between groups. The interaction protein*time represents the association between baseline protein level and change in MMSE over time, the interaction protein*time*group was entered to assess whether the association between baseline protein level and change in MMSE over time differed between groups. For interaction terms a p value of <0.10 was considered significant, for main effects p<0.05. All results are displayed as standardized B with standard error (stB[SE]).

* p<0.05; ** p<0.01; *** p<0.001 (slope different from zero)

[$] p<0.05 versus SCD/MCI+

[#] p<0.10 versus SCD/MCI -

Supplementary Table 4. Association of change in protein levels with baseline MMSE and cognitive decline

	Total cohort	SCD/MCI- (n=50)	SCD/MCI+ (n=50)	AD dementia (n=50)	$P_{interaction}$ prot*time*group
Main effect Δ SNAP-25	0.40 (1.06)				
Δ SNAP-25*time	-0.91 (0.47)[#]	0.40 (1.10)	-0.66 (0.53)	-1.22 (0.51)*	0.38
Main effect Δ SYT1	1.66 (0.75)*				
Δ SYT1*time	0.52 (0.34)				0.69
Main effect Δ NfL	-0.55 (0.58)	-0.88 (1.59)	-0.20 (0.53)	-3.44 (1.61)*[1]	
Δ NfL*time	-0.34 (0.25)	-0.22 (0.51)	-0.26 (0.19)	-1.75 (0.58)**[§$]	0.06
Main effect Δ Ng	0.73 (0.76)				
Δ Ng*time	0.26 (0.41)				0.93
Main effect Δ β-syn	0.03 (0.87)				
Δ β-syn*time	-0.69 (0.43)				0.73
Main effect Δ γ-syn	-0.24 (0.72)				
Δ γ-syn*time	-0.54 (0.34)				0.76
Main effect Δ AP2	0.95 (0.71)				
Δ AP2*time	0.43 (0.34)				0.91
Main effect Δ NPTX1	1.24 (0.88)				
Δ NPTX1*time	0.62 (0.40)				0.96
Main effect Δ NPTX2	0.79 (0.89)				
Δ NPTX2*time	0.67 (0.40)[#]				0.89
Main effect Δ NPTX-R	0.96 (0.58)				
Δ NPTX-R*time	0.25 (0.27)				0.87
Main effect Δ GDI-1	0.84 (0.66)				
Δ GDI-1*time	0.19 (0.32)				0.99
Main effect Δ PEBP1	0.43 (0.68)				
Δ PEBP1*time	0.23 (0.33)				0.98
Main effect Δ 14-3-3 ε	0.93 (0.65)				
Δ 14-3-3 ε*time	-0.37 (0.32)				0.64
Main effect Δ 14-3-3 η	0.84 (0.52)				
Δ 14-3-3 η*time	-0.10 (0.25)				0.98
Main effect Δ 14-3-3 ζ/δ	0.94 (0.77)				
Δ 14-3-3 ζ/δ*time	-0.26 (0.38)				0.67
Main effect Δ STX1-B	0.96 (0.72)				
Δ STX-1B*time	0.39 (0.33)				0.87
Main effect Δ STX-7	0.58 (0.66)				
Δ STX-7*time	0.21 (0.31)				0.85
Main effect Δ CPLX-2	0.61 (0.81)				
Δ CPLX-2*time	0.42 (0.37)				0.94

Proteins were all transformed to Z-scores and evaluated in separate models. Estimates for the total cohort were calculated without a term for group. The main effect of each protein represents the association between change in protein level and baseline MMSE, the interaction term Δ protein*group was entered to assess whether main effects of proteins differed between groups. The interaction Δ protein*time represents the association between change in protein level and change in MMSE score over time, the interaction protein*time*group was entered to assess whether the association between change in protein level and change in MMSE over time differed between groups. For interaction terms a p value of <0.10 was considered significant, for main effects p<0.05. All results are displayed as standardized B with standard error (stB[SE]).

[#] p<0.10; * p<0.05; ** p<0.01 (slope different from zero)

[$] p<0.05 versus SCD/MCI+

[§] p<0.05 versus SCD/MCI -

[1] p<0.10 versus SCD/MCI+

Supplementary Figure 1

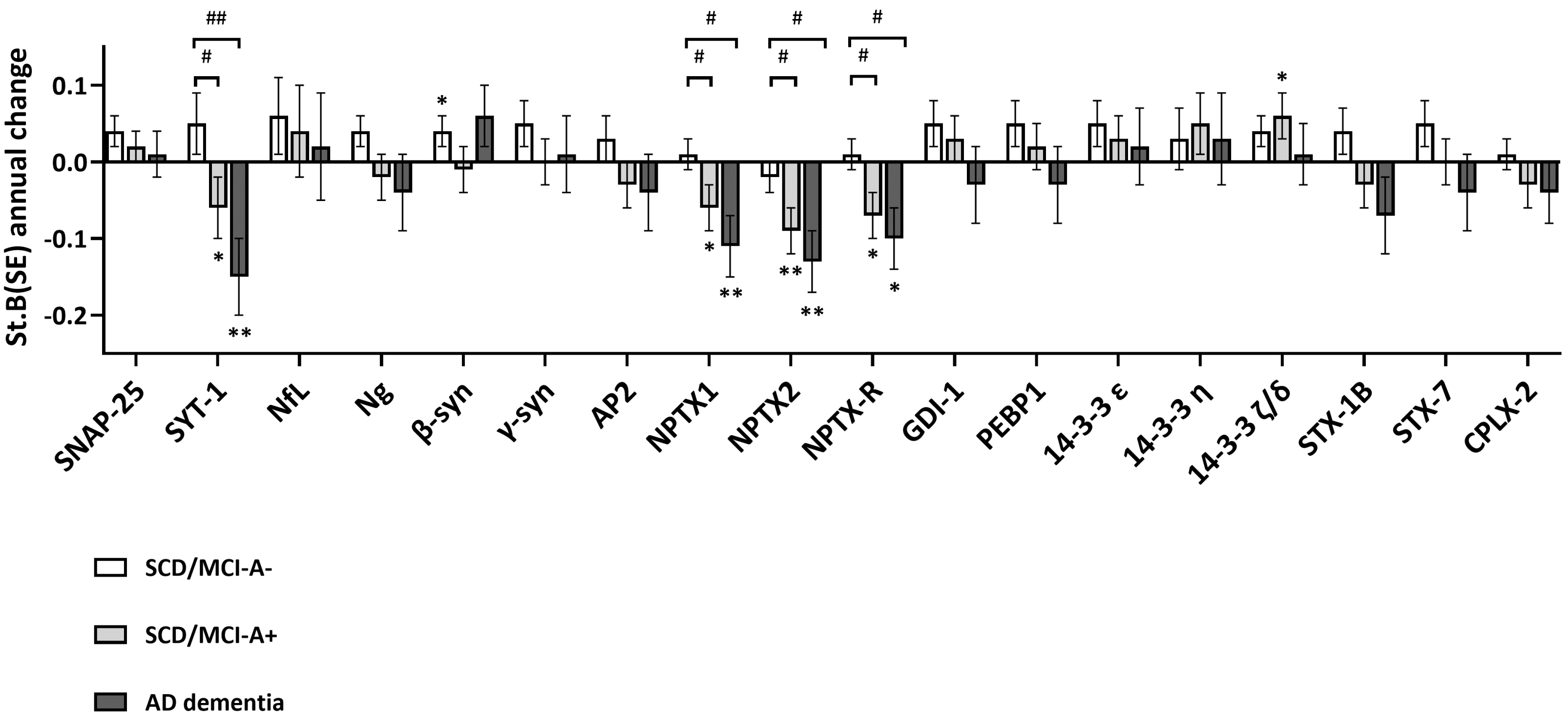

Shown are standardized betas (SE) of annual change in protein levels for all proteins, as calculated with the linear mixed model, adjusted for age, sex and MS plate (as experiments were performed on four different plates). All biomarkers were included as dependent variable in separate models, with group, time and interaction time*group as independent variables. The main effect of time represents annual change in biomarker levels, the interaction term represents differences between groups in change over time. Groups were recoded to assess main effects of time per group.

* $p<0.05$ versus slope zero; ** $p<0.01$ versus slope zero

# $p<0.05$ versus SCD/MCI-A-; ## $p<0.01$ versus SCD/MCI-A-

Supplementary Figure 2

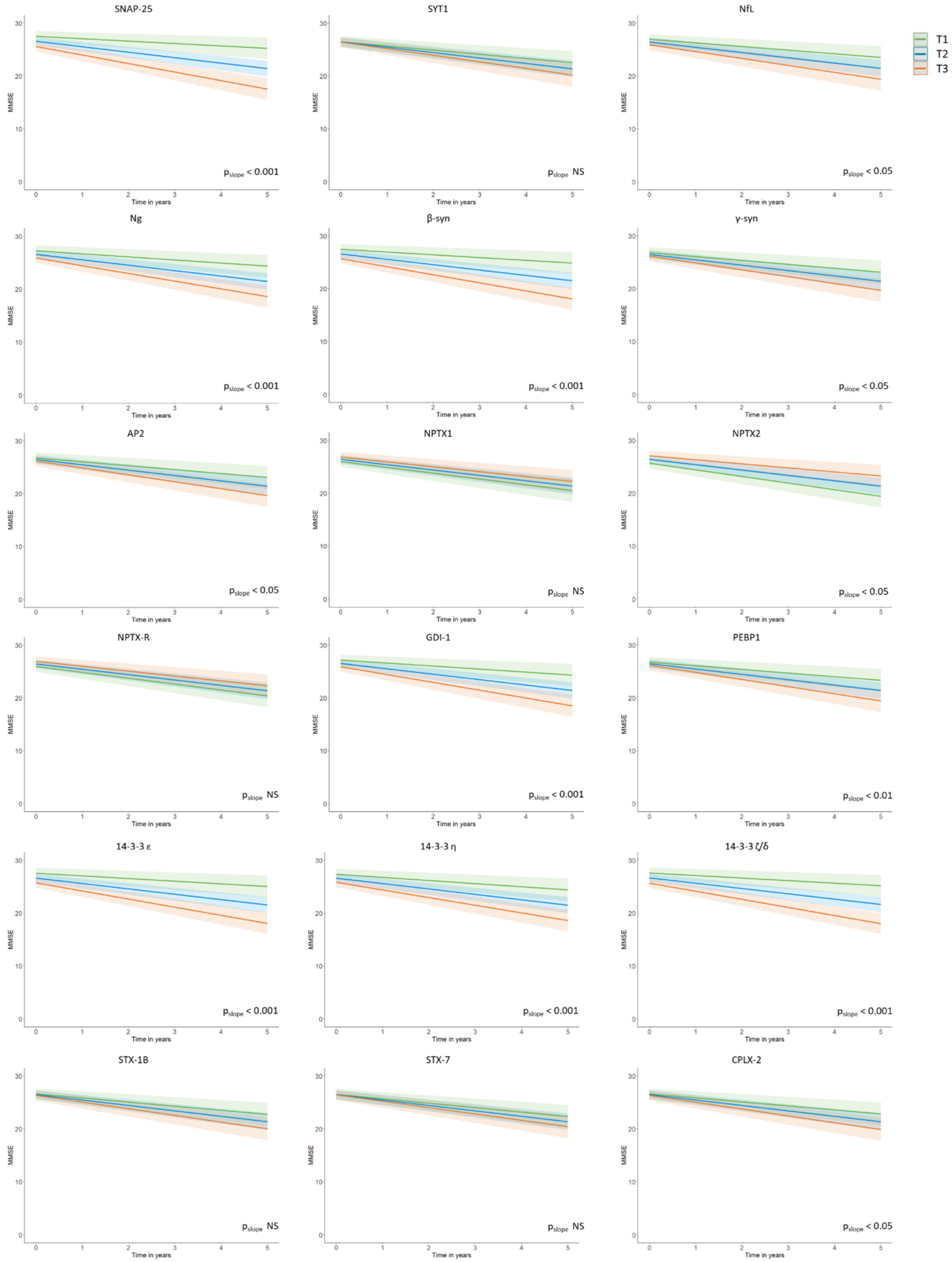

Shown are associations between proteins and cognitive decline (slopes in MMSE over time), estimated with linear mixed models. Subject specific random intercepts and random slopes with time were assumed. Age, sex and level of education were entered as covariates. Analyses were performed with continuous values for the proteins, tertiles were created for visualization purposes only.

'p_{slope}' is the p-value of the interaction protein*time.
